# Synthesis of homo- and copolymer containing sulfonic acid via atom transfer radical polymerization

**DOI:** 10.1080/15685551.2022.2126092

**Published:** 2022-09-19

**Authors:** Md. Wali Ullah, Naoki Haraguchi, Md. Azgar Ali, Md. Rabiul Alam, Samiul Islam Chowdhury

**Affiliations:** a Department of Chemistry, Faculty of Science, Comilla University, Cumilla, Bangladesh; b Department of Applied Chemistry and Life Science, Graduate School of Engineering, Toyohashi University of Technology, Aichi, Japan; c Department of Chemistry, Faculty of Science and Engineering, Bangladesh University of Textiles, Tejgaon, Dhaka, Bangladesh

**Keywords:** Atom transfer radical polymerization, well-defined homo- and copolymer, sulfonic acid functionality

## Abstract

Well-defined functional poly(*p*-phenyl styrenesulfonate) and poly(*p*-phenyl styrene-sulfonate-*co*-styrene) were successfully synthesized by the atom transfer radical polymerization (ATRP) using CuBr/bpy(PMDETA) catalyst and 1-phenylethyl bromide (1-PEBr) as an ATRP initiator in diphenyl ether (DPE) or dimethyl formamide (DMF). In both homo- and copolymers, the CuBr/PMDETA catalytic system in DPE or DME showed higher yield than CuBr/bpy and the polydispersity index (PDI) of polymer was low. Using PMDETA or bpy as a ligand in DMF, the high yield with high PDI was obtained than in DPE. We found that the CuBr/PMDETA catalyzed ATRP of *p*-phenyl styrenesulfonate and copolymerization with styrene comonomer in DPE proceeded in a controlled manner. The polymers containing sulfonic acid were obtained by the chemical deprotection of protecting group, followed by acidification. The molecular structure, molecular weights and thermal properties of the copolymers were determined by nuclear magnetic resonance (^1^H NMR) spectroscopy, Fourier transform infrared (FT-IR) spectroscopy, size exclusion chromatography (SEC), differential scanning calorimetry (DSC) and thermogravimetric analysis (TGA), respectively.

## 1. Introduction

The controlled radical polymerizations (CRPs) such as nitroxide-mediated radical polymerization (NMP) [1,2], atom transfer radical polymerization (ATRP) [3–8], reversible addition-fragmentation chain transfer (RAFT) polymerization [9–11], etc. have been used as synthetic tools to prepare well-defined polymers or copolymers with predetermined molecular weights, precise chain-end functionalities, and controlled topologies. ATRP has become a promising one of the different CRPs due to its mild reaction conditions, broad applicability to a variety of monomers, and good control over the polymer chain length and chain-end functionalities [3,4,12–36]. In ATRP, the molecular weights of the resultant polymer can be calculated using the ratio of monomer to the initiator in the polymer chain. The different tailor-made polymers with complex architectures like block copolymers, star polymers, dendrimers and hyperbranched polymers can be synthesized by varying the composition and topology of the polymer chains [37–42].

Homo- and copolymers of styrene sulfonate [43] have much interest in the field of renewable energy. The sulfonated polymers were widely used as hole transporters in organic solar cells [44,45], ionic exchangers in polymer-lithium batteries [46,47], proton conductors in fuel cells [48,49], and as heterogenous acid catalysts in the organic reactions such as esterification, olefin hydration, etherification and alkylation of phenols [50–54].

Coughlin and coworkers have reported the sulfonation of polystyrene (PS) and the emulsion polymerization of styrene sulfonate (SS) monomer [55]. They found that small shoulders appeared in the ^1^H NMR spectrum of poly(styrene sulfonate) (PSS) obtained through sulfonation. The shoulders were not observed in PSS synthesized by polymerizing of styrene sulfonate (SS) monomer. This result indicated that the side reactions such as incomplete sulfonation, sulfonation in different positions, or other side products during sulfonation of PS were occurred.

The ATRP of monomers with sulfonic acids are difficult because of their strong reacting with either the ATRP catalyst, alkyl halide‑type initiator or the polymeric dormant species [56]. In these cases, the sulfonic acid group in a monomer can be protected as an alkyl ester, salts formed at high pH or by neutralization with alkyl amines before the polymerization. Examples of protective groups include *t*‑butyl, benzyl, tetrahydropyrannyl, 4‑nitro-phenyl, and 1‑ethoxyethyl [25,57]. The protecting groups can control the polymer properties [58] and the obtained polymers can be more functionalized by transformation reaction of these groups.

There have been few reports on the synthesis of PSS and copolymerization of SS with a comonomer by the controlled radical polymerizations. Mitsukami et al. have prepared homopolymer PSS and block copolymer with sodium 4-vinylbenzoate in aqueous media by RAFT [43]. They observed that block copolymer was pH responsive.

To find out a suitable ATRP condition is very important for the polymerization of a monomer especially with a polar functional group. In our previous work, we reported the ATRP conditions for the homoplymerization of styrene and acrylates [59].

In the present work, we have successfully synthesized well-defined sulfonated homopolymers (**PSS**) and copolymers (**CP-SS**) with chain-end functionalities by the ATRP of *p*-phenyl styrene-sulfonate (SS) and copolymerization with styrene (S) comonomer, respectively. The effects of ATRP ligand and solvent on the polymer yields, molecular weights (*M*_n_), and polydispersity index (PDI) were investigated in detail. These polymers were characterized by Fourier transform infrared spectroscopy (FT-IR), nuclear magnetic spectroscopy (^1^H NMR) and size exclusion chromatography (SEC). Thermal properties of the copolymers were investigated by the thermogravimetric analyses (TGA) and differential scanning calorimetry (DSC).

## 2. Experimental

### 2.1. Materials and instrumentation

In this study, styrene monomer (S), copper bromide (CuBr) and solvents [diphenyl ether (DPE) and dimethyl formamide (DMF)] used were purified according to a standard procedure. The ATRP ligands (2,2’-bipyridine (bpy), and *N,N,N’,N”,N”-*pentamethyldiethylenetriamine (PMDETA)) and 1-phenylethyl bromide (1-PEBr) as an initiator were used as received without further purification. The ^1^H NMR of polymers were measured in CDCl_3_ at 25 °C operating at 400 MHz using the JEOL JNM-ECS 400SS spectrometer and the chemical shits were expressed in δ ppm. FT-IR spectra were recorded with a JEOL JIR-7000 FT-IR spectrometer and reported in reciprocal centimeter (cm^−1^). The molecular weights and polydispersity index (PDI) of polymers were determined by size exclusion chromatography (SEC) with Tosoh instrument with HLC 8020 UV (254 nm) detection. DMF was used as a carrier solvent at a flow rate of 1.0 mL.min^−1^ at 40 °C. Two polystyrene gel columns of bead size 10 μm (Shodex KF-806 L, Showa Denko K. K.) were used. Differential scanning calorimetry (DSC) analysis was performed on an SII EXSTER 600 (Seiko Instruments Inc., Japan) system under a nitrogen atmosphere. Differences in the thermal history of the polymers were eliminated by first heating the specimen to 200 °C, cooling it to 20 °C, and then recording the second DSC scan (all heating rates: 10 °C/min). Thermal gravimetric analysis (TGA) was carried out using a TG/DTA 6000 analyzer (Seiko Instruments Inc., Japan) under a flow of nitrogen (constant heating rate: 10 °C/min; 25–550 °C).

### 2.2. Synthesis of p-phenyl styrenesulfonate (SS) monomer

*p*-Phenyl styrenesulfonate (SS) was synthesized according to the previously reported procedure [60,61]. Yield: 3.082 g, 65%, *R*_f_ = 0.54. ^1^H NMR (400 MHz, CDCl_3_, *δ *= 7.27 (CDCl_3_), TMS): *δ* = 5.48 (d, *J* = 10.7 Hz, 1H), 5.91 (d, *J* = 17.7 Hz, 1H), 6.76 (dd, *J* = 11.0 and *J* = 17.7 Hz, 1H), 6.99 (d, *J* = 7.9 Hz, 2 H; Ar-H), 7.24–7.31 (m, 3 H; Ar-H), 7.51 (d, *J* *= *8.2 Hz, 2 H; Ar-H), 7.77 (d, *J* = 7.0, 2 H; Ar-H). ^13^C NMR (100 MHz, CDCl_3_, *δ *= 77.1 (CDCl_3_), TMS): *δ* = 118.35, 122.37, 126.67, 127.16, 128.85, 129.65, 129.88, 134.03, 135.10, 143.27, 149.59.

### 2.3. Synthesis of functional poly(p-phenyl styrenesulfonate) PSS by ATRP

CuBr (14 mg, 0.098 mmol), SS (1302 mg, 5.000 mmol), and 1.25 mL of DPE were added to a 6 mL vial successively. The reaction mixture was purged with argon for 5 min and then PMDETA (52 mg, 0.30 mmol) was added. After an additional 5 min of argon bubbling, 1-PEBr (19 mg, 0.10 mmol) as an ATRP initiator was added into the reaction mixture. The molar ratio of [SS]_o_/[1-PEBr]_o_/[CuBr]_o_/[PMDETA]_o_ was set to 100/2/2/6. The polymerization was performed in an oil bath at 110 °C for 24 h with a stirring rate at 400 rpm. The polymers were precipitated by dropwise addition in 125 mL of methanol. The **PSS** was collected by filtration and then dried at 40 °C under vacuum oven. The molecular structure and *M*_n_ of **PSS** were determined by ^1^H NMR spectroscopy and SEC, respectively. Yield: 67%; *M*_n, th_ = 8,910, *M*_n, SEC_ = 12,900, *M*_n, NMR_ = 8,780, *M*_w_/*M*_n_ = 1.31; FT-IR (KBr): *ν* = 1374, 1181 (S = O stretching), 1600, 1489 and 1452 (C = C in aromatic ring), 3060, 3025 (C – H in aromatic ring), and 2925, 2850 (C – H in alkyl) cm^−1^.

### 2.4. Synthesis of functional poly(p-phenyl styrene-sulfonate-co-styrene) CP-SS by ATRP

CuBr (15 mg, 0.10 mmol), 70 mol% of S (366 mg, 3.51 mmol), 30 mol% of SS (390 mg, 1.50 mmol), and 1.25 mL of DPE were added to a 6 mL vial successively. The reaction mixture was purged with argon for 5 min and then PMDETA (54 mg, 0.31 mmol) was added. 1-PEBr (20 mg, 0.11 mmol) as an initiator was added into the mixture after an additional 5 min of argon bubbling. The polymerization was carried out in an oil bath at 110 °C for 24 h with a stirring at 400 rpm. The resultant copolymers were precipitated by dropwise addition in 125 mL of methanol. The **CP-SS** was collected by filtration and then dried at 40 °C under vacuum oven to provide a white powder. Yield: 45%; *M*_n, th_ = 8,390, *M*_n, SEC_ = 15,600, *M*_n, NMR_ = 9,230, *M*_n_/*M*_w_ = 1.53; FT-IR (KBr): *ν* = 1376, 1176 (S = O stretching), 1598, 1490 and 1453 (C = C in aromatic ring), 3059, 3024 (C – H in aromatic ring), and 2924, 2851 (C – H in alkyl) cm^−1^.

### 2.5. Synthesis of PSSNa and CP-SSNa

**General synthesis procedure**: To a flask with a magnetic stir bar inside, three equivalents of NaOH to SS moiety in the polymer or copolymer was added in a mixed solvent (THF/MeOH/H_2_O (50/10/1) = 16.8/3.36/0.336) was added. The reaction was conducted in an oil bath at 50 °C for 24 h.

**PSSNa**: After 24 h, the reaction mixture was cooled to room temperature, and the insoluble fraction was collected by centrifugation and washed with THF, MeOH, and CH_3_COCH_3_. The solid product **PSSNa** was dried at 40 °C for 24 h under vacuum oven. Yield: 89%; SSNa content: 4.78 mmol g^−1^; FT-IR (KBr): *ν* = 1189 (S = O stretching in SO_3_Na), 1646, 1601 and 1450 (C = C in aromatic ring), 3060 (C – H in aromatic ring), 2924, 2848 (C – H in alkyl), 3465 cm^−1^ (O – H).

**CP-SSNa**: The resultant polymers were precipitated by dropwise addition in ether (Et_2_O). The insoluble fraction was collected by centrifugation and washed with a small amount of MeOH and CH_3_COCH_3_. The solid product **CP-SSNa** was dried at 40 °C for 24 h under vacuum oven. Yield: 90%; SSNa content: 2.18 mmol g^−1^; FT-IR (KBr): *ν* = 1190 (S = O stretching in SO_3_Na), 1602, 1490 and 1450 (C = C in aromatic ring), 3060, 3024 (C – H in aromatic ring), 2925, 2850 (C – H in alkyl), 3464 cm^−1^ (O – H).

### 2.6. Synthesis of CP-SSH

To a flask with a magnetic stir bar, **CP-SSNa** (459 mg, 0.100 mmol of SSNa moiety) and 53 mL of THF were added. 1.10 mL (20 equivalents to SSNa moiety) of the diluted H_2_SO_4_ in THF was added slowly into the mixture. The reaction was performed at room temperature for 24 h. The polymers were precipitated by dropwise addition in Et_2_O. The insoluble fraction was collected by centrifugation and washed with a small amount of MeOH and CH_3_COCH_3_. The solid product was dried at 40 °C for 24 h under vacuum oven. Yield: 96%; sulfonic acid (SSH) moiety content: 2.30 mmol g^−1^; FT-IR (KBr): *ν* = 1218 (S = O stretching in SO_3_H), 1602, 1490, 1450 (C = C in aromatic ring), 3060, 3024 (C – H in aromatic ring), and 2924, 2850 (C – H in alkyl).

## 3. Results and discussion

### 3.1. Synthesis of poly(p-phenyl styrene-sulfonate) PSS by ATRP

**Scheme 1.**
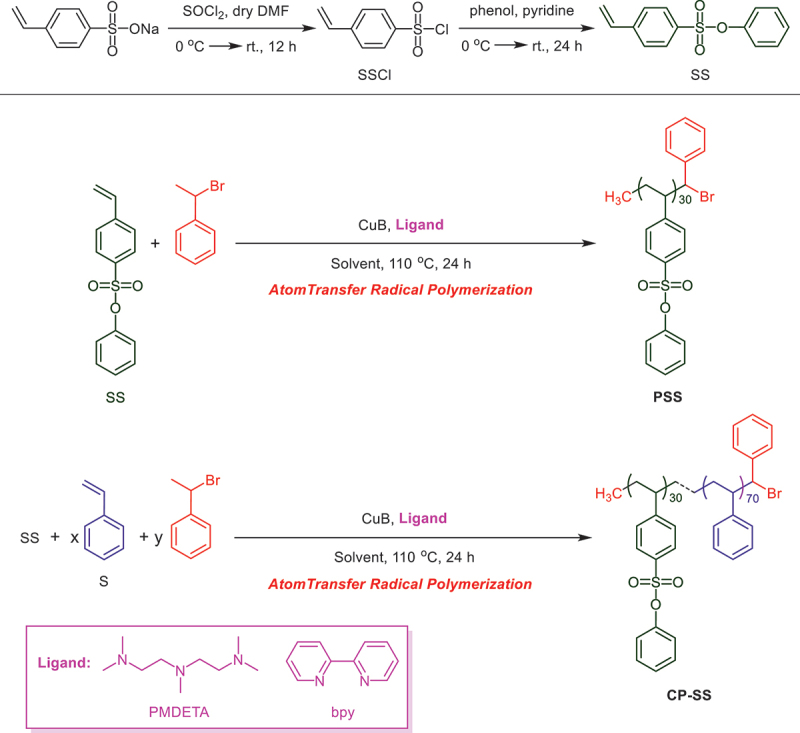
Synthesis of **PSS** and **CP-SS.**

**PSS** was prepared by the ATRP of SS, as illustrated in Scheme 1. The effects of ligand and solvent on the polymer yield and the polydispersity were summarized in Table 1. The ATRP conditions for SS were set as same as S polymerization [59]. **PSS** was obtained in low yield than **PS** because of the bulky functional group (C_6_H_5_OO_2_S –) present in the SS monomer (entry I vs. 1; entry II vs. 2). On the other hand, the *M_n_* of **PSS** with high PDI compared to **PS** were higher than the calculated because of the chain termination at the early stage of polymerization. S polymerization showed high PDI values when PMDETA was used as an ATRP ligand instead of bpy, probably due to the more initiating species formed firstly (entry I vs. II), whereas SS polymerization exhibited low polydispersity (entry 1 vs. 2). DPE was changed to DMF for checking the solvent effect on the ATRP of SS. The yield of **PSS** was increased when DMF with bpy or PMDETA was used as a solvent in lieu of DPE and the PDI values were relatively high (entry 1 vs. 3; entry 2 vs. 4). A similar rate enhancement in polar media was observed from different studies [62]. A faster initiation might have occurred due to high solubility of catalyst and polar monomer in DMF. The ATRP of SS with PMDETA in DPE or DMF proceeded faster than bpy and the PDI values were also low (entry 1 vs. 2; entry 3 vs. 4).

**Table 1.** Characterization data for **PSS** and or **CP-SS** synthesized by ATRP.^a.^

Entry	Polymer	Ligand	Solvent	Yield (%)	*M*_n,th_	*M*_n,SEC_^b^	*M*_n,NMR_^c^	*M*_w_/*M_n_^b^*	Molar ratio (S:SS)^c^
I*	PS	PMDETA	DPE	81	4,400	4,470	4,240	1.27	-
1^d^	PSS	PMDETA	DPE	67	8,910	12,900	8,780	1.31	-
II*	PS	bpy	DPE	54	3,000	3,070	2,980	1.06	-
2^d^	PSS	bpy	DPE	50	6,690	12,500	13,200	1.44	-
3^d^	PSS	PMDETA	DMF	74	9,810	13,700	11,400	1.42	-
4^e^	PSS	bpy	DMF	58	7,730	12,800	13,500	1.45	-
5^e^	CP-SS	PMDETA	DPE	45	8,390	15,600	9,230	1.53	70:30
6^e^	CP-SS	bpy	DPE	26	4,920	5,570	3,790	1.10	50:30
7^e^	CP-SS	PMDETA	DMF	51	9,470	17,100	10,350	1.79	60:30
8^e^	CP-SS	bpy	DMF	37	6,920	17,500	12,500	2.26	50:30

[Monomer]_o_ = 4.0 M, [1-PEBr]_o_ = [CuBr]_o_ = 0.08 M, and [bpy(PMDETA)]_o_ = 0.24 M. ^a^Polymerizations were performed at 110 °C for 24 h. [M]_o_/[1-PEBr]_o_/[CuBr]_o_/[bpy(PMDETA)]_o_ = 100/2/2/6. *M*_n, th_ = [{(MW)_M_ × Conversion × ([M]_o_/[1-PEBr]_o_)} + (MW)_1-PEBr_]. ^b^Determined by SEC using DMF as an eluent at a flow rate of 1.0 mL.min^−1^ at 40 °C (polystyrene standards). ^c^Determined by ^1^H NMR spectroscopy. ^d^SS was used as a monomer. ^e^S and SS were used as monomers. * [59]

**Figure 1.**
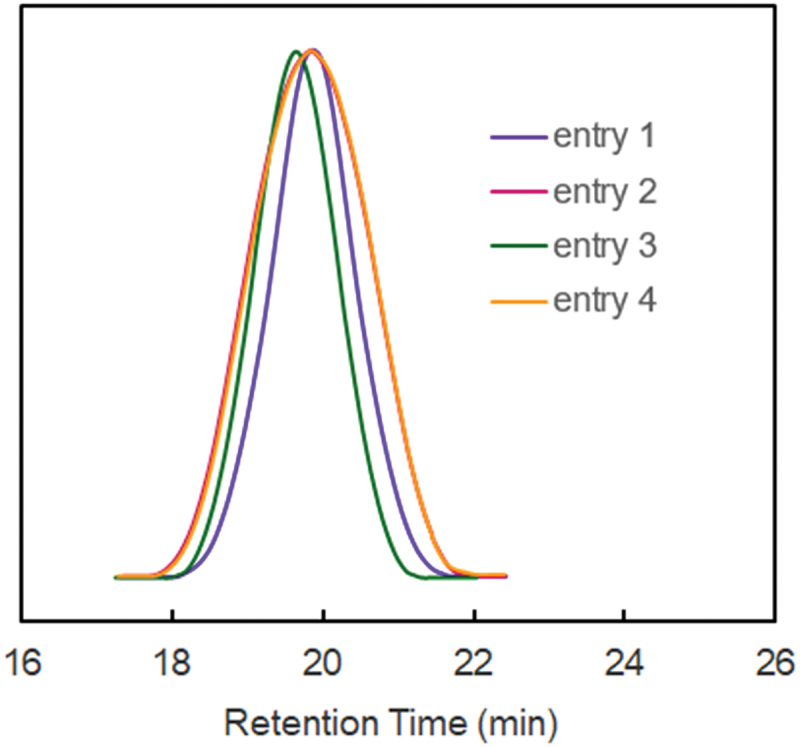
SEC traces of **PSS.**

Figure 1 shows the SEC traces of **PSS** synthesized by the ATRP using CuBr/ bpy(PMDETA) catalytic system of SS in DPE or DMF. In the SEC traces, the molecular weight distributions (MWD) of **PSS** were moderate and unimodal (PDI1.5).

**Figure 2.**
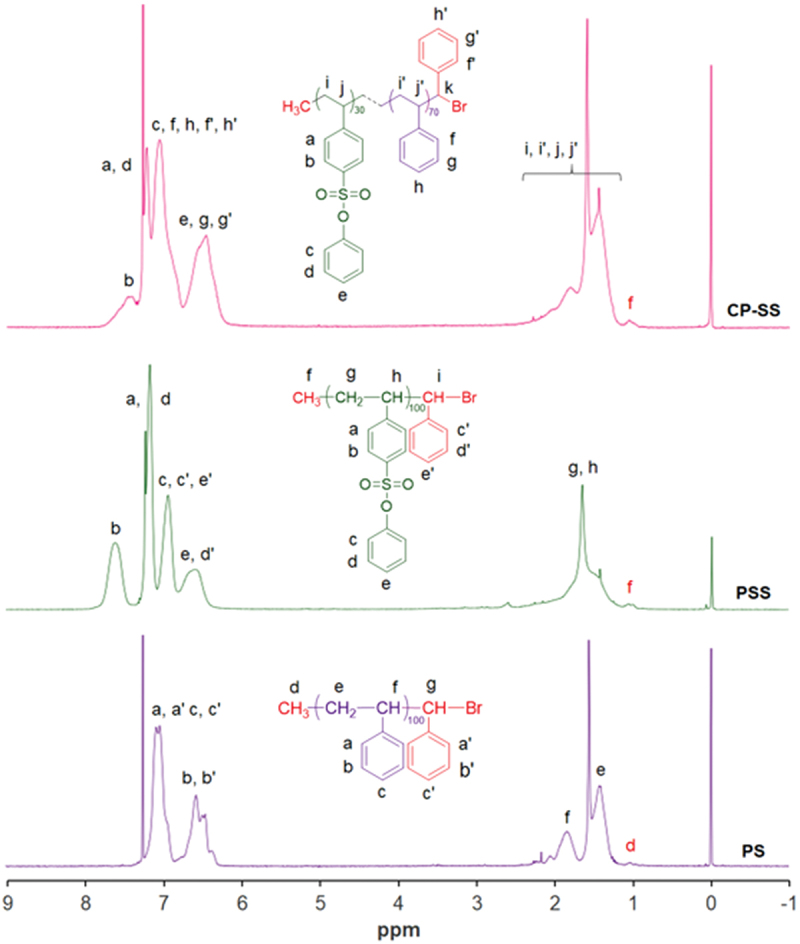
^1^H NMR spectra of **PS, PSS**, and **CP-SS** in CDCl_3._

**Figure 3.**
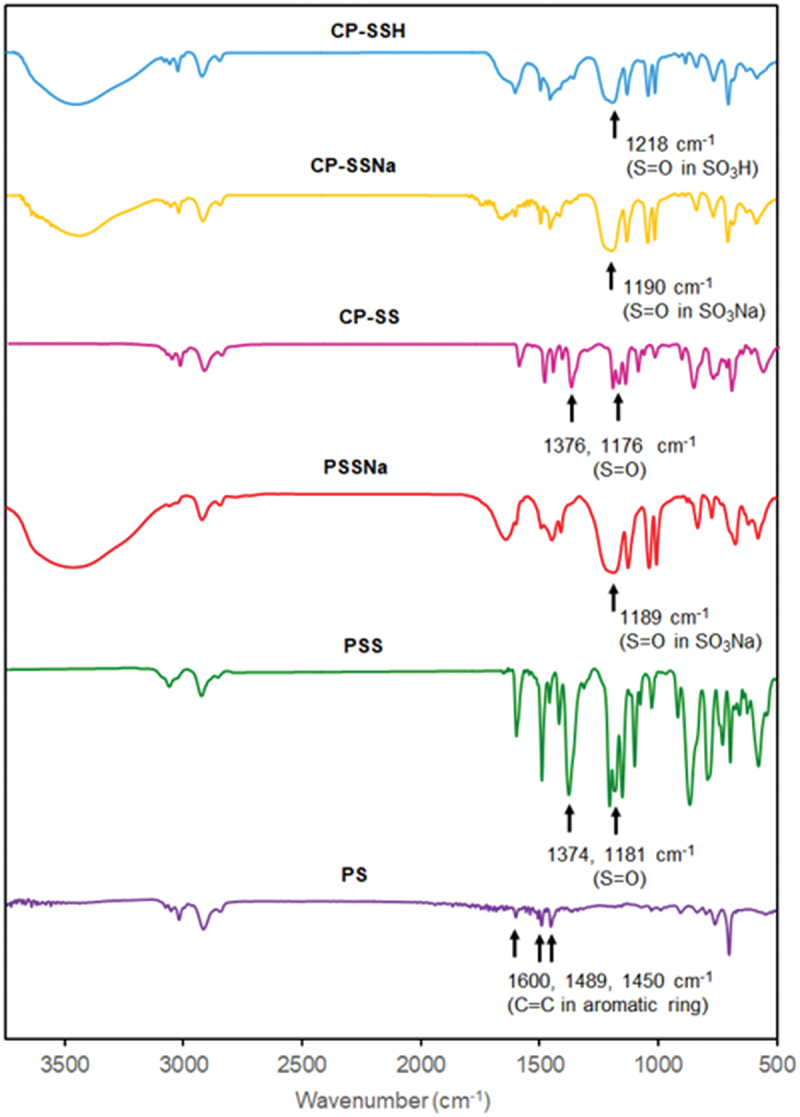
FTIR spectra of **PS, PSS, PSSNa, CP-SS, CP-SSNa**, and **CP-SSH.**

The structure of **PSS** was characterized by ^1^H NMR and FT-IR spectra. From the ^1^H NMR spectrum of **PSS** in Figure 2, the peaks appeared at 1.3–1.8 ppm and 6.5–7.7 ppm confirmed the present of aliphatic and aromatic protons, respectively. The peak positions of aromatic protons in **PSS** chains were shifted to down field than in **PS** due to the electronic effect of sulfonate group. In FT-IR spectrum of **PSS** in Figure 3, the typical stretching bands for C = C bonds in aromatic rings were found at 1560, 1489 and 1452 cm^−1^. The asymmetrical and symmetrical stretching vibration peaks for S = O bond were observed at 1374 (strong) and 1181 (weak) cm^−1^, respectively.

These results indicated that well-defined function-nal **PSS** successfully synthesized by the CuBr/ PMDETA catalyzed ATRP in DPE.

### 3.2. Synthesis of poly(p-phenyl styrene-sulfonate-co-styrene) CP-SS by ATRP

The properties of **PS** can be regulated by the copolymerization with a commoner [63,64]. After the successful synthesis of **PSS, CP-SS** was prepared by the copolymerization of 30 mol% SS with 70 mol% S using the same ATRP conditions that was employed in **PSS**, as illustrated in Scheme 1.

When ATRP was employed using bpy or PMDETA as a ligand in DPE or DMF, the yield of **CP-SS** was lower than **PSS** because ATRP catalyst may also coordinate with S = O of the SS comonomer which reduced the catalytic activity (entries 5–8 vs. 1–3). The *M*_n_ of **CP-SS** was higher than the calculated. The *M*_n_ was in good agreement with *M_n_*,_th_ when bpy was used in DPE. Copolymerization of SS and S with PMDETA in DMF showed slightly high yield than in DPE. It may be possible that the polarity of DMF increases the solubility of catalysts and monomers that might have assisted the initiation. By contrast, high *M*_n_ was obtained due to the chain termination at the end of polymerization and the PDI (=1.79) values were also high. The rate of copolymerization was also increased when bpy was used in DMF and the *M*_n_ of **CP-SS** with high polydispersity (PDI = 2.26) was high probably owing to the chain termination at the end of polymerization.

**Figure 4.**
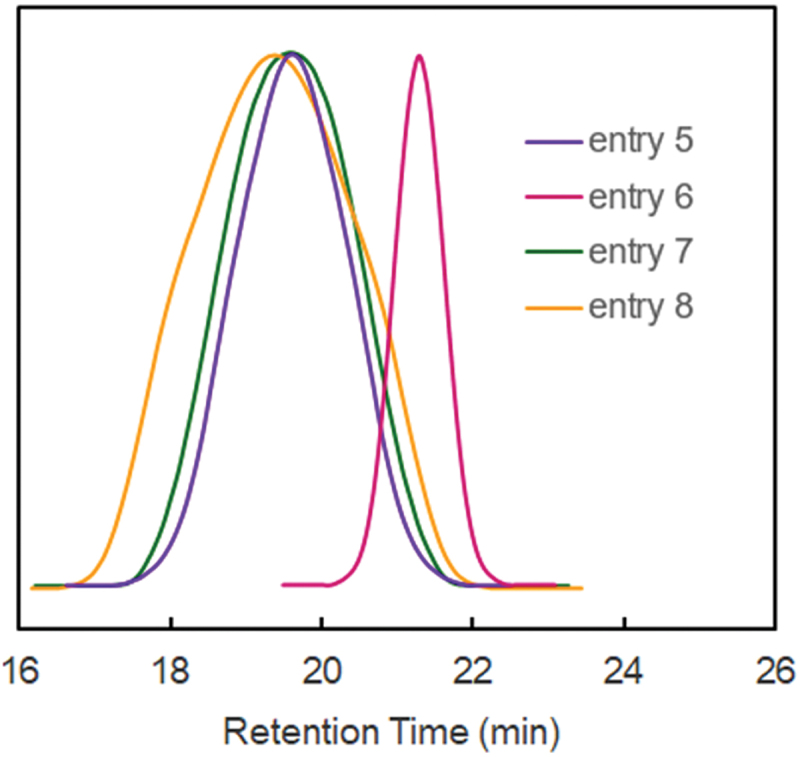
SEC traces of **CP-SS.**

Figure 4 exhibits the SEC traces of **CP-SS** prepared by the CuBr/bpy(PMDETA) catalyzed ATRP of SS with S in DPE or DMF. In SEC traces, the MWDs of **CP-SS** were broader when bpy or PMDETA was used in DMF than in DPE (entries 7 and 8).

In ^1^H NMR spectrum of **CP-SS** in Figure 2, the peaks for aliphatic and aromatic protons of SS and S moieties in **CP-SS** chains were observed at 1.3–1.8 ppm and 6.5–7.5 ppm, respectively, which were found in **PSS**. The peak appeared at 1.10 ppm confirmed the present of chain-end methyl protons from 1-PEBr initiator in the **PSS** and **CP-SS** chains. The molar ratio of SS and S measured from the ^1^H NMR spectrum was 60:30 that was close to the calculated one.

In FT-IR spectrum of **CP-SS** in Figure 3, the asymmetrical and symmetrical stretching vibrational bands for S = O bond were found at 1176 and 1376 cm^−1^, respectively, that were exhibited in **PSS**. These results indicated that copolymerization of SS with S was successfully proceeded.

These above results confirmed that well-defined **CP-SS** was successfully synthesized using the suitable ATRP conditions as same as the SS polymerization.

***3.3. Synthesis of poly(styrenesulfonate-co-styrene) CP-SSNa and poly(styrene sulfonic acid-co-styrene) CP-SSH by deprotection***

**Scheme 2.**
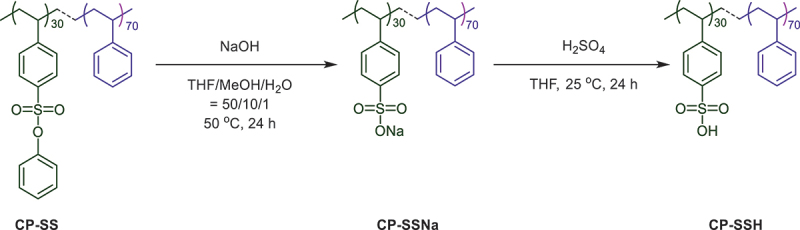
Synthesis of **CP-SSH.**

**PSSNa** or **CP-SSNa** was obtained by the deprotect-tion of phenyl group in the SS moiety of polymer chain using three equivalents NaOH to SS moiety of **PSS** or **CP-SS** in a mixed solvent (THF/MeOH/H_2_O = 50/10/1) at 50 °C for 24 h (Scheme 2). The yield and SSNa content in **PSS** and **CP-SS** were 89%, 4.78 mmol g^−1^ and 90%, 2.18 mmol g^−1^, respectively.

The broad absorption peaks found at 1189 cm^−1^ and 1190 cm^−1^ in the FT-IR spectra of **PSSNa** and **CP-SSNa** (Figure 3), respectively, confirmed the S = O bond in SO_3_Na in both the homopolymer and copolymer. **CP-SSH** was prepared by treating of twenty equivalents conc. H_2_SO_4_ to SSNa moiety of **CP-SSNa** in THF. The yield and sulfonic acid content were in quantitative. FT-IR of **CP-SSH** in Figure 3 exhibited that broad absorption band assigned to stretching vibration of S = O bond in SO_3_H. This result confirmed that **CP-SSH** was successfully prepared from the deprotection of **CP-SS**, followed by acidification.

### 3.4. Thermal properties

**Figure 5.**
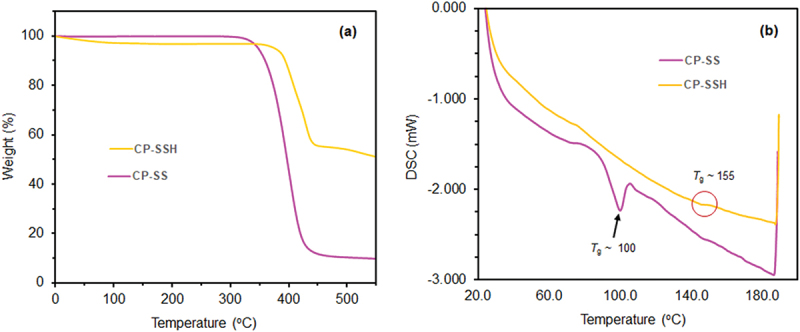
(a) TGA and (b) DSC thermograms of **CP-SS** and **CP-SSH.**

The thermal properties of **CP-SS** and **CP-SSH** were studied by TGA and DSC analysis. TGA and DSC thermograms for **CP-SS** and **CP-SSH** are shown in Figures 5(a,b). TGA of **CP-SS** showed that the degradation temperatures (*T*_d_) of **CP-SS** (345 °C) were very close to *T*_d_ of polystyrene (PS) [65]. The *T*_d_ of **CP-SSH** were shifted to higher temperatures as a function of sulfonic acid (– SO_3_H) groups, as compared to PS and **CP-SS** (from 345 °C to 375 °C). In endothermic DSC thermograms, the similar results were observed i.e., the glass transition temperatures (*T*_g_) of **CP-SSH** were also shifted to higher temperatures due to the presence of polar – SO_3_H groups in the polymer chains, as compared to PS and **CP-SS** (from 100 to 155 °C). The high *T*_g_ values of **CP-SSH** can be considered as an indicator of the increase of structural rigidity of **CP-SSH** chains.

## 4. Conclusion

Well-defined functional poly(*p*-phenyl styrene-sulfonate) **PSS** and poly(*p*-phenyl styrenesulfonate-*co*-styrene) **CP-SS** were successfully synthesized by the CuBr/bpy(PMDETA) catalyzed atom transfer radical polymerization (ATRP) of *p*-phenyl styrene-sulfonate (SS) and copolymerization with styrene (S) comonomer using 1-PEBr as an initiator in DPE or DMF. The effects of ligand and solvent on the polymer yield, the molecular weights (*M*_n_), and the polydispersity (PDI) were investigated. In **PSS** and **CP-SS**, the CuBr/PMDETA catalytic system in DPE or DME showed higher yield than CuBr/bpy and the polydispersity of polymer was low. Using PMDETA or bpy as a ligand in DMF, the high yield was obtained than in DPE and the PDI values were high. We found that the CuBr/PMDETA catalyzed ATRP of SS and copolymerization of SS with S in DPE proceeded in a controlled manner. **CP-SSH** containing sulfonic acid was obtained by the chemical deprotection of protecting group, followed by the acidification reaction. The molecular structure and molecular weights of the polymers were determined by nuclear magnetic resonance (^1^H NMR) spectroscopy, Fourier transform infrared (FT-IR) spectroscopy and size exclusion chromatography (SEC), respectively. **CP-SSH** copolymers showed higher degradation temperatures with high *T*_g_ values, as compared to PS and **CP-SS**.
